# A Chinese Foundling Asylum

**Published:** 1900-06

**Authors:** 


### A CHINESE FOUNDLING ASYLUM.

One of the greatest of Chinese wickednesses, and one which missionaries are constantly deploring, is child-murder. Foundlings are frequently left at the door of that magnificent Italian institution, commonly called the Girls' Orphanage, at Hankow. The institution now consists of five blocks of main buildings two stories high. The number of inmates is seldom less than 1,000, and up to the present time 94,650 taels (£12,000) have been spent on the grounds and buildings. The expenses amount to about 200,000 francs per annum, and this is contributed mainly by France and Italy. Females of all ages are admitted to the orphanage, from a baby to an old woman; though the institute prefers to have them when they are young. The inmates come in many different ways, and for many different reasons. The babies are received much in the same way as in all foundling hospitals. Baskets filled with hay are placed on the veranda at the front door of the orphanage, and the mothers who bring the children deposit them in the baskets. There is always a Chinese watchwoman on the lookout for these waifs of humanity, and as soon as they are noticed they are taken in and attended to.

Notwithstanding all the attention bestowed upon them, the mortality among the foundlings is very high, and if 90 out of 100 live it is considered very good. Even after babyhood is past the foundlings require constant care and attention, and have to be treated with cod-liver oil and maltine until they are five or six years old. And this, of course, means that had they been left to the tender care of their Chinese mothers not one of them would have lived. It is a well-known fact that the parentage of female Chinese children who die is enormous. Girl-babies are not a desideratum in a Chinese household, so that when too many come along they are gotten rid of. Parents who are not devoid of all natural feelings bring their children to the orphanage and leave them there. On several occasions babies only one day old have been left in the baskets; and it is no great wonder that such frail morsels of humanity succumb to the effects of such unnatural treatment. There are no fewer than 500 nurses in the institution. All kinds of curious precautions are taken to prevent the babies from getting mixed up, each child having a special collar fastened around its neck.—*The Wide World*, London, May, 1900.

